# Molecular structure-property correlations from optical nonlinearity and thermal-relaxation dynamics

**DOI:** 10.1016/j.cplett.2008.12.064

**Published:** 2009-02-03

**Authors:** Indrajit Bhattacharyya, Shekhar Priyadarshi, Debabrata Goswami

**Affiliations:** a Department of Chemistry, Indian Institute of Technology, Kanpur 208 016, India; b Centre for Laser Technology, Indian Institute of Technology, Kanpur 208 016, India

## Abstract

We apply ultrafast single beam Z-scan technique to measure saturation absorption coefficients and nonlinear-refraction coefficients of primary alcohols at 1560 nm. The nonlinear effects result from vibronic transitions and cubic nonlinear-refraction. To measure the pure total third-order nonlinear susceptibility, we removed thermal effects with a frequency optimized optical-chopper. Our measurements of thermal-relaxation dynamics of alcohols, from 1560 nm thermal lens pump and 780 nm probe experiments revealed faster and slower thermal-relaxation timescales, respectively, from conduction and convection. The faster timescale accurately predicts thermal-diffusivity, which decreases linearly with alcohol chain-lengths since thermal-relaxation is slower in heavier molecules. The relation between thermal-diffusivity and alcohol chain-length confirms structure-property relationship.

## Graphical abstract

A molecular structure-property relationship is established from the variation of third-order nonlinear susceptibility and thermal-diffusivity for a primary alcohol series.

## 1 Introduction

Infrared lasers are a powerful tool for investigating molecular vibrations. In this Letter, we use pulsed IR lasers to study the nonlinear optical response in primary alcohols. Our results show a correlation between the structure of the alcohol and their nonlinear optical coefficients.

Typically, optical nonlinearities are measured at very dilute concentrations, e.g., at ∼10^−4^ M, under the assumption that the non-interacting solvents essentially do not contribute to the effective nonlinearity of the solute. However, structural modifications of organic solvents can have profound effects on nonlinear properties. An earlier example has been the sensitive acoustic experiments with primary alcohols [1,2] that measured the pressure–density nonlinearity [3,4] as the acoustic characteristics of the media, changed with the chain-length of the alcohols [1]. In our nonlinear measurements with femtosecond lasers, as well, the enhanced sensitivity when the thermal effects are managed has lead us to the useful correlation of molecular structure with the nonlinear properties of the primary alcohols.

There is considerable interest in the study of two-photon absorption coefficients [5,6], saturation absorption coefficients (*β*) [7–9], and nonlinear index of refraction (*n*_2_) in materials at 1560 nm because of their potential applications in optoelectronics, photonics devices and material science. We study *β* and *n*_2_ for a homologous series of primary alcohols as our model system. This model system enables us to establish a structure-property relationship by monotonically increasing the chain-length of the alcohols and measuring their corresponding *β* and *n*_2_.

Several studies on optical nonlinearity in materials through Z-scan technique [6,10] have been reported. Wavelength dependence of third-order susceptibilities have also been explored both theoretically [11] and experimentally [12]. Previous study of optical nonlinearity variations in alcohol molecules differing in size and structure were reported at 780 nm wavelength [13] where the extremely small two-photon cross-sections essentially reflected changes in *n*_2_. However, as we establish here, studies with high repetition-rate laser experiments without chopper had thermal corruption.

## 2 Method

We use open and close aperture Z-scan experiments, in analogy to the saturation absorption work discussed earlier in water [8], to respectively measure the *β* and *n*_2_ for a series of primary alcohols with the help of 1560 nm femtosecond laser pulses, however, with the important inclusion of an optical-chopper. The vibrational combination states of the alcohols are coupled by the femtosecond laser pulses at 1560 nm. These couplings result in the absorption of 1560 nm and the excited molecules undergo relaxation through non-radiative processes, which gives rise to transient thermal effects. These transient thermal effects are related to the pure optical nonlinearity of the samples and can be measured as a change in their *n*_2_ values [14]. The transient thermal effects of individual pulses accumulate in case of high repetition-rate lasers to produce a cumulative thermal effect at longer timescales. We measure this cumulative thermal effect with the mode-mismatched two-color pump–probe experiment.

We remove the cumulative thermal effect for high repetition-rate laser experiments with the help of an optical-chopper to recover pure optical nonlinearity. When we use an optical-chopper, its off-time window provides sufficient time for thermal-relaxation, and we acquire all our measurements immediately with the chopper opening [15]. However, thermal-relaxation variation for different samples and their correlation with their molecular structures is yet to be attempted. So, we explore this correlation by the thermal lens (TL) spectroscopy, which is highly attractive, nondestructive, and noninvasive. In many cases, TL spectroscopy is the most sensitive optical technique for measuring the thermal properties of solids and liquids [16,17].

Thermal lens can be treated as an ideal thin concave lens of a particular focal length that causes distortion of light passing through it and this simple model has been studied in detail [18–20]. Many authors have used pump–probe experiments and two-color Z-scan techniques to measure TL in different samples [8,21–23]. In our specific experiments, we use two collinear femtosecond laser pulses at 1560 nm as the pump and at 780 nm as the probe, to explore the evolution of thermal-relaxation timescales in different samples. The choice of pump–probe beam diameters is important for such experiments. For example, while modeling the aberrant nature of TL through pump–probe scheme under steady state condition, Shen et al. [24,25] used a higher probe beam-diameter to include larger area information, since thermal excitation perturbs not only the thermal nature within the excitation beam radius but also outside it. However, the thermal-relaxation dynamics from this model would wrongly show that different points in the sample experience different thermal-relaxation rates due to the existence of different thermal gradients in their vicinity. In order to circumvent such difficulties, we use smaller probe beam-diameter in our experiments, so that it is totally contained within the thermally excited region in the sample. Our probe beam radius, in particular, was chosen to be half the size of that of the pump beam. The variation in the timescales has also been used to get information about molecular structure-property correlation.

## 3 Experiment

**Fig. 1:**
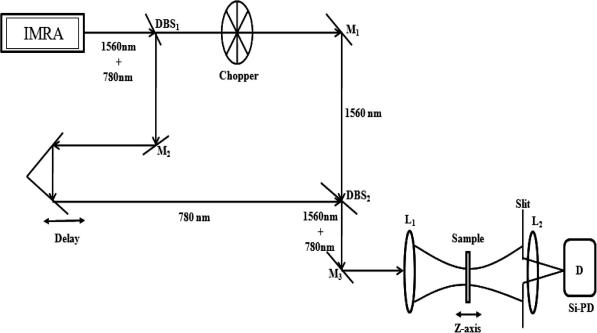
Schematic of the experimental set up: DBS = Dichroic Beam Splitter, M = Mirror, L = Lens, D = Detector.

Our experimental setup (Fig. 1 ) involves a mode-locked femtosecond Er:doped fiber laser (Femtolite, IMRA Inc.), which generates pulses centered at fundamental wavelength 1560 nm and its second harmonic 780 nm collinearly as a single beam with 50 MHz repetition-rate. The 1560 nm pulse is ∼300 fs wide and has an average power of 40 mW while the 780 nm pulse is 90 fs wide and has an average power of 20 mW. A collinear Mach–Zhendar interferometer is built with the help of two dichroic beam splitters: one separates the two wavelengths and the other recombines them.

**Fig. 2:**
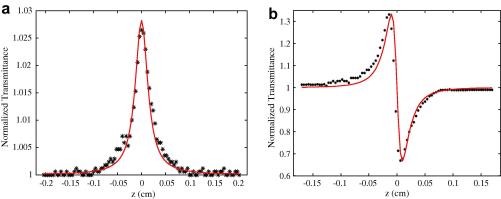
(a) Z-scan transmittance trace: open aperture fit in case of methanol and (b) Z-scan transmittance trace: close aperture fit in case of methanol.

For the single beam third-order optical nonlinear measurements at 1560 nm, we block the 780 nm arm in the interferometer and allow only the 1560 nm pulses whose beam-diameter is 5 mm. We used 15 mW of 1560 nm laser power, which resulted in the maximum peak-power of 7.97 × 10^8^ W/cm^2^ at the focal point in the samples. After passing through the sample, the transmitted beam is focused with another focusing lens into the Peltier cooled InGaAs photodiode (Acton Research), and the corresponding signal is measured with a 500 MHz oscilloscope (LeCroy LT354M) interfaced to computer with a National Instruments GPIB card. We measure *β* and *n*_2_ of alcohols from standard open and closed aperture Z-scans respectively. We take the samples in 1 mm thick quartz cuvette. A motorized translation stage (model ESP-300), which can step with a minimum resolution of 0.0554078 μm, moves the sample across the focal point of the first lens and data acquisition is performed using LabView programming. HPLC-grade alcohols were obtained from Sigma–Aldrich and were used without further purification. The requisite purity of these samples was confirmed through their individual UV–vis spectra. The Z-scan transmittance for open aperture (saturation absorption) and closed aperture (nonlinear-refraction) are plotted using Matlab (Fig. 2 a and b).

For measurements of the thermal-relaxation process in liquids, 1560 nm and 780 nm beams are simultaneously focused into the sample cell after passing through the collinear interferometer (Fig. 1). The 1560 nm beam, which acts as the thermal pump in our experiment, is chopped with an optical-chopper having 50% duty cycle. A silicon photo-detector (Thorlab: DET210) is used to detect the 780 nm probe beam through an 80% closed aperture to determine the thermal-relaxation. The chopping of the 1560 nm beam provides on–off modulation to the thermal pump. Different chopper frequencies provide the correspondingly different thermal ‘on’ and ‘off’ times to the sample. We measure the response to this thermal effect with the 780 nm probe beam as the chopper frequencies are varied from 3 Hz to 350 Hz.

## 4 Results and discussion

In the third-order optical nonlinearity measurements at 1560 nm, we measure the normalized transmittance versus the sample position for both closed and open aperture cases and determine the values of *n*_2_ and *β*, respectively. We have used the phenomenological model [6] for fitting our data though we are aware of the other molecular state fitting models [26,27] that are also possible. Since we focus on the trends of nonlinear coefficients rather than the molecular energy state mechanisms, the phenomenological fits that we use are sufficiently reliable and are widely accepted in literature. The *β* values are obtained by curve fitting the observed open aperture transmittance, *T*(*z*), which is measured as a function of sample position with respect to the focal point as per the following Eq. (6):

(1) $$T(z\text{,}S=1)=\overset{\infty }{\underset{m=0}{\sum }}\frac{{[-q_{0}(z\text{,}0)]}^{m}}{{(m+1)}^{\frac{3}{2}}}$$

where,

(2) $$q_{0}(z\text{,}t)=\frac{\beta I_{0}(t)L_{\mathrm{eff}}}{\left(1+\frac{z^{2}}{z_{0}^{2}}\right)}$$

Here *T*(*z*, *S* = 1) is the normalized transmittance, such that, *S* = 1 denotes the open aperture case, *β* is the nonlinear absorption coefficient, *I*_0_(*t*) is the peak on-axis irradiance at the focus, *L_eff_* = (1 − *e^αL^*)/*α* is used for the induced third-order nonlinearity, *L* is thickness of the cuvette, *α* is linear absorption coefficient and *z*_0_ is the Rayleigh range = π*ω*_0_/*λ*. The materials that exhibit absorption saturation show a decrease in their absorption coefficient when measured using high laser intensity. The absorption coefficient *α* depends on the intensity *I* of the incident laser as:

(3) $$\alpha =\frac{\alpha_{0}}{1+\frac{I}{I_{S}}}$$

where *α*_0_ is the low-intensity absorption coefficient, and *I*_S_ is a parameter known as the saturation intensity. In the saturation absorption process, the third-order optical response is due to the population change induced by single photon absorption. The resulting modified population interacts with the field and thus, the overall process results in no net photon absorption.

On the other hand, the *n*_2_ is determined from close aperture Z-scan traces using the following fitting Eq. (6):

(4) $$T(z\text{,}\Delta \varphi_{0})=1+\frac{4\Delta \varphi_{0}x}{\left(x^{2}+9)(x^{2}+1\right)}$$

where, *x* = *z*/*z*_0_, Δ*φ*_0_(*t*) = *k*Δ*n*_0_(*t*)*L*_eff_, *k* = wavevector = 2π/*λ*, *λ* is wavelength, Δ*n*_0_(*t*) = *n*_2_ *I*_0_(*t*), *n*_2_ is the coefficient of nonlinear-refraction in m^2^/W.

The *β* and *n*_2_ are respectively related to the imaginary and real parts of the third-order susceptibility as follows [6]:

(5) $$\text{Im}(\chi^{\left(3\right)})=\frac{n_{0}\varepsilon_{0}c^{2}\beta }{\omega }$$

(6) $$\text{Re}(\chi^{\left(3\right)})=2n_{0}^{2}\varepsilon_{0}{\mathrm{cn}}_{2}$$

where *n*_0_, as before, is the linear refractive index, *ε*_0_ is the permittivity of space, *c* is the velocity and *ω* is the angular frequency of light.

The absolute value of *χ* ^(3)^, i.e. |*χ* ^(3)^|, for all the samples was calculated from the following relationship:

(7) $$|\chi^{\left(3\right)}|={\left[{\left(\text{Re}(\chi^{\left(3\right)})\right)}^{2}+{\left(\text{Im}(\chi^{\left(3\right)})\right)}^{2}\right]}^{1/2}$$

Subsequent to such high repetition-rate femtosecond laser pulse excitation, the molecules undergo de-excitation through non-radiative pathways, which often induces long transient thermal lensing in the samples. Measure of nonlinear optical parameters for the sample is thus corrupted by thermal effects. To measure pure optical nonlinear parameters, the thermal effect should be removed. We noticed in our experiments that the thermal effect can be effectively probed through close aperture Z-scan measurements. Furthermore, we show that it is easier to remove thermal effect in open aperture Z-scan as compared to the close aperture experiments with the help of an optical-chopper.

We systematically explore the removal of thermal effect by using methanol as a representative case. We used an optical-chopper and measured the peak-power of 1560 nm in both the open and close aperture situations as the chopper-frequency was gradually changed from 5 Hz to 500 Hz with 50% duty cycle. This corresponded to an on–off-time range of 100–1 ms. The use of the chopper does not change or modulate the repetition-rate of the laser and the number of pulses striking the sample per second remains same. However, the number of pulses that actually strike the sample in a row before being turned off reduces by a factor of 100 (i.e., changes from 5 × 10^6^ pulses to 5 × 10^4^ pulses) as our chopper-frequency increases from 5 Hz to 500 Hz. In addition, as the chopper-frequency increases, the chopper opening rise-time correspondingly reduces and quickly approaches the limit where it is remarkably smaller than the thermal-relaxation time of the sample, and since we only measure the maximum height of the 1560 nm signal, the better the rise-time of the chopper opening, the better is the removal of the thermal effect.

**Fig. 3:**
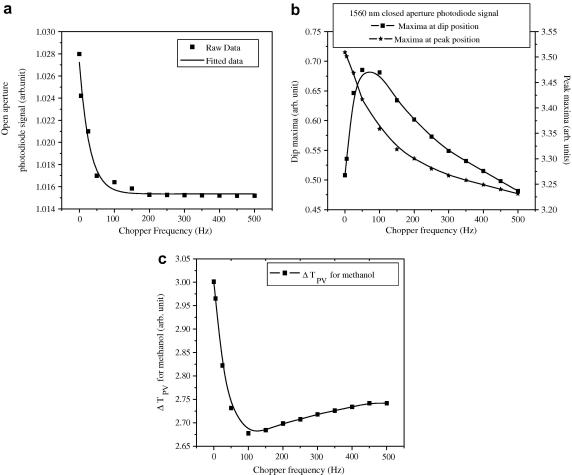
(a) Change in the maximum in the 1560 nm photodiode signal with different chopper frequencies in open aperture case (in case of methanol), (b) change in the maximum in the photodiode voltage in dip position and maximum in the photodiode voltage in peak position with different chopper frequencies in close aperture case (in case of methanol) and (c) variation of Δ*T*_PV_ with different chopper frequencies in case of methanol.

For the open aperture experiments with 1560 nm laser, we positioned our samples at the lens focus, which also corresponded to the minimum position (dip) of the Z-scan trace and changed the frequency of the chopper. The chopper-frequency modulated waveforms were directly recorded from the oscilloscope and the maximum in the InGaAs photodiode voltage was monitored for each chopper-frequency. These maxima values were then plotted with respect to the individual chopper frequencies (Fig. 3a). In the closed aperture case, two situations arose (Fig. 3b). In one case, we kept our sample at the minimum position (dip) while in the other case we kept our sample at the maximum position (peak) of the close aperture Z-scan traces. We observed the variation in the maximum of the photodiode voltage with chopper-frequency in both cases, which resulted in the two plots in Fig. 3 b.

From this study, we find that the peak-power becomes almost constant for open aperture case after 250 Hz, indicating the removal of thermal effect, though it is not as obvious in the close aperture case. However, the close aperture case also has important embedded information, which is revealed when we plot the peak to dip separation (Δ*T*_PV_) with respect to individual chopper frequencies as in Fig. 3c. In fact, the maximum values measured at the peak and dip positions of the close aperture Z-scan (Fig. 3b) for different chopper frequencies behave oppositely at low chopper frequencies to effectively minimize thermal effect. Since Δ*T*_PV_ is proportional to the *n*_2_, it essentially reduces with increasing chopper-frequency to reach the value of pure *n*_2_ as the thermal effect becomes reduced at higher chopper frequencies. The minimum position in Fig. 3c is also important, which we discuss later.

**Fig. 4:**
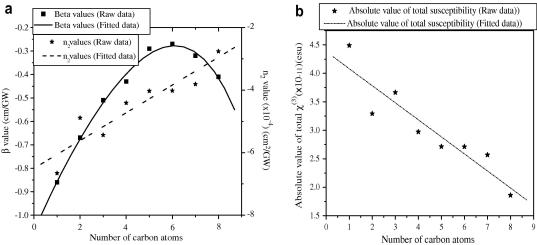
(a) Variation of nonlinear absorption coefficient (*β*) and nonlinear refractive index (*n*_2_) with the change in the number of carbon atoms in the alcohol series and (b) variation of total third-order nonlinear susceptibility (*χ*^(3)^) with the change in the number of carbon atoms in the alcohol series.

As discussed above for single beam experiments at 1560 nm wavelength, all alcohols in the series show non-resonant saturation absorption and cubic nonlinear-refraction. The saturation effect is expected due to the vibronic transition at this particular wavelength. In both the cases, we get negative values of *β* and *n*_2_. In Fig. 4 a we show that with the increase of chain-length, i.e., with the increase in the number of carbon atoms in homologous primary alcohols, there is a gradual linear increase in *n*_2_, whereas the variation of the *β* values shows a parabolic increase with the maximum value at hexanol. We also monitored the variation in the absolute value of total third-order susceptibility, i.e., |*χ* ^(3)^| with increasing number of carbon atoms in the alcohol series (Fig. 4b), which shows that total |*χ* ^(3)^| decreases linearly as the chain-length increases along the alcohol series.

Thermal excitation of samples induces cylindrical equi-temperature surfaces in the samples, which have the same axis as that of the excitation laser beam. The innermost temperature surface has the highest temperature resulting in the lowest refractive index and *vice versa*, which in turn generates a graded index waveguide like condition in the sample that manages to misguide the beam from its path. As a consequence, for a two-beam pump–probe experiment, a hole is burnt at the centre of the probe beam in its spatial profile after passing through a thermally excited medium. The radius of the burnt hole depends on the temperature gradient of the probed region. When the chopper blocks the pump beam, thermal-relaxation takes place and the temperature gradient in the probed region decreases, which is directly proportional to the thermal-relaxation. This results in a reduction in the radius of hole in the probe beam. Thus, a measurement of the increase in the probe beam power (in mV) through the close aperture Z-scan gives an effective estimate of this thermal-relaxation process.

**Fig. 5:**
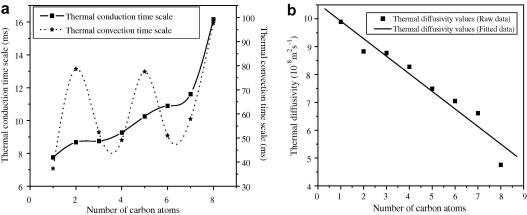
(a) Variation of thermal conduction and thermal convection time scales with the change in the number of carbon atoms in the alcohol series and (b) variation of thermal-diffusivity with the change in the number of carbon atoms in the alcohol series.

In order to measure thermal-relaxation for alcohols, we use 1560 nm and 780 nm as the pump and the probe beams, respectively. In presence of 1560 nm pump, we perform close aperture Z-scans for each sample and detect only the probe beam (780 nm) transmittance by a silicon photo-detector. We keep the sample at the dip (minimum) position of the close aperture Z-scan traces and measure the variation of 780 nm probe power with the modulation of the chopper-frequency in the pump beam. In all our measurements, we take an average of at least 500 data points, so that, the standard deviation is within 1%. We plot the variation of the 780 nm probe power with respect to different chopper-frequency for every sample. Next, we fit them with exponential decay models, which enable us to determine the timescales associated with their respective relaxation. We find that, for all the samples, the probe power through the close aperture decreases bi-exponentially with increase in the chopper-frequency, implying that the thermal-relaxation is a bi-exponential process involving two timescales. For such non-radiative thermal-relaxation processes, bi-exponential decay means that it cannot be explained with a pure conduction process only. So, we attribute the faster decay time, *τ*_1_, to the thermal conduction process and the slower decay time, *τ*_2_, to thermal convection. Both *τ*_1_ and *τ*_2_ were found to have an overall increasing trend that can be correlated with the variation of molecular structures of the alcohols along the series. An oscillating behavior is also embedded in this overall increase, more drastic in case *τ*_2_, which we find has a correlation to the density variation across the alcohol series (Fig. 5 a).

Thermal conduction timescale (*τ*_1_) can also be expressed as follows [28–33]:

(8) $$\tau_{1}=(\rho C/\kappa )\cdot \omega_{e}^{2}$$

(9) $$\text{or}\;\tau_{1}=\omega_{e}^{2}/D$$

where *C*, *ρ* and *κ* are the specific heat, density and thermal conductivity of the samples, respectively. *D* is the sample’s thermal-diffusivity and *ω_e_* is the pump beam radius in the sample. In our case, the beam waist of 1560 nm at the focal point of the lens is found to be 27.7 μm. From the above equations (Eqs. (8) and (9)), we calculate the thermal diffusivities for each of the alcohols using the time constants we determined experimentally. The diffusivity values for all the alcohols are reported in Table 1 . The trend in the thermal-diffusivity values are shown in Fig. 5b, which shows that thermal-diffusivity values decrease gradually as the chain-length increases along the alcohol series. As the chain-length increases, the size of the molecule becomes larger resulting in slower relaxation timescale for the higher members of the alcohols.

We find that the values of the thermal-diffusivity that we determined for the alcohol series are in agreement with the previously reported values for methanol and ethanol [34]. Finally, we would also like to point out that, for the case of methanol, the minimum position of ∼125 Hz in Fig. 3c corresponds to the thermal conduction timescale of ∼8 ms in Fig. 5a, which, in fact, provides us an alternate route to measure thermal conduction timescales from simple single beam Z-scan measurements. Besides methanol, this could be generalized to other samples as well. Removal of thermal effect as was discussed earlier (∼250 Hz for methanol), therefore, represents a minimum chopper-frequency which is roughly twice the thermal-relaxation time of the sample concerned.

## 5 Conclusion

We have shown how molecular structures can be correlated to their optical nonlinear response by calculating their total third-order optical nonlinear susceptibility from a respective measure of *β* and *n*_2_ values of a series of primary alcohols with standard single beam open and close aperture Z-scan experiments. All nonlinear measurements were performed using 1560 nm femtosecond light pulses. The molecular structure-property correlation shows that the *β* values follow a parabolic increase, whereas the *n*_2_ values increase almost linearly as the primary alcohol chain-length increases. In contrast, |*χ* ^(3)^| decreases linearly as the alcohol chain becomes longer along the series. All these sensitive nonlinear studies have become possible as we managed to minimize the thermal effect with the use of an optical-chopper with optimized frequency.

Our investigations with thermal lens pump–probe technique resulted in measurements of thermal-relaxation timescales and thermal-diffusivity of the homologous primary alcohol series. Two thermal-relaxation timescales were observed experimentally for such non-radiative process: a faster component, *τ*_1_ corresponding to thermal conduction and a slower, *τ*_2_, representing thermal convection, both of which show an increasing trend for longer chain alcohols. We calculate thermal-diffusivity values from the experimental *τ*_1_ values, which, in general, decrease linearly with chain-length of the alcohols as expected, since thermal-relaxation is sluggish in heavier molecules. The diffusivity values agree with the previously reported theoretical and experimental values in literature. We also show an alternate measure of thermal conduction timescales from simple single beam Z-scan measurements instead of the mode-mismatched two-color pump–probe Z-scan experiments. Our work, thus, provides the generalization that a minimum chopper-frequency of roughly twice the thermal-relaxation time of the sample concerned is sufficient for the removal of thermal effect.

## Figures and Tables

**Table 1:** Diffusivity for different alcohols as compared to previous available data.

Sample	Diffusivity (×10^−8^ m^2^/s)	Theoretical diffusivity [34]	Values reported in separate experiment [34]
Methanol	9.89	9.98	10.1
Ethanol	8.83	8.78	8.65
Propanol	8.77	∗	∗
Butanol	8.28	∗	∗
Pentanol	7.49	∗	∗
Hexanol	7.05	∗	∗
Heptanol	6.61	∗	∗
Octanol	4.75	∗	∗

‘∗’ is not yet reported in the literature.
